# Tackling Inherited Blindness

**DOI:** 10.1371/journal.pmed.0020402

**Published:** 2005-11-01

Imagine the eye is like a camera. The shutter, like the iris of the eye, opens and closes to let in the right amount of light. The lens helps focus light on the film. And the film is like the retina. Regardless of the quality of the camera, if the film is faulty, the developed pictures may be distorted or blurred. In this way, untreatable degenerative diseases of the retina, which affect millions of people worldwide, lead to varying degrees of irreversible blindness. These degenerative eye disorders include retinitis pigmentosa, which affects 1.5 million people, and age-related macular degeneration, which is a leading cause of blindness in North America. The list of inherited retinal dystrophies (degenerations) is long and includes Best disease, choroideremia, cone–rod dystrophy, congenital stationary night blindness, and Leber congenital amaurosis (LCA).

LCA is a collection of diseases all characterized by severe loss of vision at birth from retinal dysfunction. It is a leading cause of congenital blindness. Currently, there is no treatment for LCA; however, it is known that LCA can be caused by mutations in the gene encoding RPE65, a key protein involved in the production and recycling of the chromophore 11-*cis*-retinal (11-*cis*-RAL) in the eye. 11-*cis*-RAL is an integral part of rhodopsin and cone visual pigments, pigments essential for our vision. About 15% of patients with LCA have mutations in *RPE65*. Humans with this form of LCA and Rpe65-deficient mice models both have severely impaired rod and cone function.

Armed with this knowledge, scientists are honing in on various therapeutic strategies for genetic eye diseases. These strategies include somatic gene therapy, infusion of protective proteins, and embryonic cell transplantation. The hope is that such interventions will converge and lead to treatments that slow down or prevent the blindness characteristic of many degenerative eye diseases. [Fig pmed-0020402-g001]

**Figure pmed-0020402-g001:**
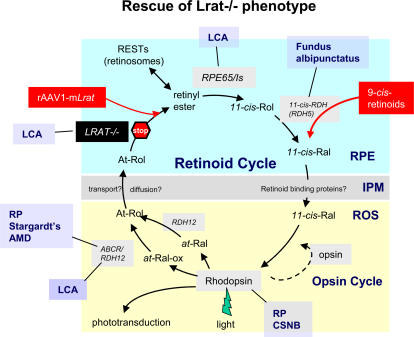
Pharmacological and rAAV Gene Therapy Rescue of the Retinoid Cycle

There have been several attempts to restore vision in patients with LCA using interventions such as calcium channel blockers and intraocular injection of neurotrophic factors. In most cases, the effects of these treatments lasted less than a month; hence, repeated administrations were required. Another approach is to bypass the biochemical block in mice without functional Rpe65 using synthetic *cis*-retinoids administered orally; such treatments have induced dramatic improvement in photoreceptor physiology.

Also, somatic gene therapy has been very successful in many animal models of retinal degeneration. In this issue of *PLoS Medicine*, Krzysztof Palczewski and colleagues attempted to combine two approaches to restore visual function with intraocular gene therapy and oral pharmacologic treatment with novel retinoid compounds in lecithin retinol acyl transferase (LRAT)–deficient mice. LRAT is a key enzyme involved in storage of vitamin A in the form of retinyl esters in structures known as retinosomes. In mice without LRAT, no 11-*cis*-RAL chromophore is produced, and visual function is severely impaired. *Lrat* mutations have been detected in a subset of patients with LCA.

The team found that gene therapy using intraocular injection of recombinant adeno-associated virus carrying the *Lrat* gene successfully restored electroretinographic and pupillary light responses in *Lrat^−/−^* mice. Production of 11-*cis*-RAL was also restored. Pharmacological intervention with orally administered pro-drugs 9-*cis*-retinyl acetate and 9-*cis*-retinyl succinate also caused long-lasting restoration of retinal function in *Lrat*-deficient mice. Combining interventions produced markedly increased levels of visual pigment, and 1,000-fold improvements in pupillary light response and electroretinogram sensitivity. Direct comparison of each treatment was difficult, but both therapies provide efficient recovery of higher order visual responses. One advantage of oral retinoid treatment was its ease of administration compared with the subretinal injections required for viral vectors. Another factor was that the orally administered compounds were not stored in the liver for long, and were quickly oxidized and secreted. Pharmacological treatment could also be given multiple times; several low-dose treatments show cumulative effects. The main disadvantage of oral treatment was the potential for long-term systemic toxicity compared with vector targeting of LRAT to the RPE, which needs to be examined in future studies.

Interestingly, the researchers observed that chromophore supplementation and somatic gene therapy were optimally effective in combination, particularly when chromophore supplementation was continued at low doses for longer periods of time. The authors suggest that the combined approach might be more suitable for treating a wider age range of patients. Although much more preclinical testing is required, it is likely that pharmacologic and somatic gene therapeutic approaches could be used together if such testing proves safe and successful in human trials. The authors speculate that treatment of patients with oral retinoids could begin in infancy to avoid amblyopia while also avoiding the difficulties associated with surgery in very young patients. For older patients, a long-lasting drug-free treatment might be achieved by surgical introduction of viral vectors.

